# Genotypic Diversity and Drug Susceptibility Patterns among *M. tuberculosis* Complex Isolates from South-Western Ghana

**DOI:** 10.1371/journal.pone.0021906

**Published:** 2011-07-11

**Authors:** Dorothy Yeboah-Manu, Adwoa Asante-Poku, Thomas Bodmer, David Stucki, Kwadwo Koram, Frank Bonsu, Gerd Pluschke, Sebastien Gagneux

**Affiliations:** 1 Noguchi Memorial Institute for Medical Research, University of Ghana, Legon, Ghana; 2 University of Bern, Institute for Infectious Diseases, Bern, Switzerland; 3 Ghana Health Services, Accra, Ghana; 4 MRC, National Institute for Medical Research, London, United Kingdom; 5 Swiss Tropical and Public Health Institute, Basel, Switzerland; 6 University of Basel, Basel, Switzerland; Institut Pasteur de Lille, France

## Abstract

**Objective:**

The aim of this study was to use spoligotyping and large sequence polymorphism (LSP) to study the population structure of *M. tuberculosis* complex (MTBC) isolates.

**Methods:**

MTBC isolates were identified using standard biochemical procedures, *IS6110* PCR, and large sequence polymorphisms. Isolates were further typed using spoligotyping, and the phenotypic drug susceptibility patterns were determined by the proportion method.

**Result:**

One hundred and sixty-two isolates were characterised by LSP typing. Of these, 130 (80.25%) were identified as *Mycobacterium tuberculosis* sensu stricto *(*MTBss*),* with the Cameroon sub-lineage being dominant (N = 59/130, 45.38%). Thirty-two (19.75%) isolates were classified as *Mycobacterium africanum* type 1, and of these 26 (81.25%) were identified as West-Africa I, and 6 (18.75%) as West-Africa II. Spoligotyping sub-lineages identified among the MTBss included Haarlem (N = 15, 11.53%), Ghana (N = 22, 16.92%), Beijing (4, 3.08%), EAI (4, 3.08%), Uganda I (4, 3.08%), LAM (2, 1.54%), X (N = 1, 0.77%) and S (2, 1.54%). Nine isolates had SIT numbers with no identified sub-lineages while 17 had no SIT numbers. MTBss isolates were more likely to be resistant to streptomycin (p<0.008) and to any drug resistance (p<0.03) when compared to *M. africanum*.

**Conclusion:**

This study demonstrated that overall 36.4% of TB in South-Western Ghana is caused by the Cameroon sub-lineage of MTBC and 20% by *M. africanum* type 1, including both the West-Africa 1 and West-Africa 2 lineages. The diversity of MTBC in Ghana should be considered when evaluating new TB vaccines.

## Introduction

Despite the World Health Organisation declaring tuberculosis (TB) a global emergency in 1993, TB remains a major global health problem. About 9 million new TB cases and 2 million deaths occur each year. TB is the leading cause of adult mortality caused by a single infectious agent worldwide [1]–[3]. Similar to other countries in sub-Saharan Africa, TB is a major public health problem in Ghana. In 2004, it was estimated that the prevalence of all forms of TB was 376/100,000, with an annual incidence of 206 cases per 100, 000 populations. The annual risk of infection with TB was estimated to be between 1–2%; deaths due to TB averaged 50/100,000 annually [1].

The backbone of TB control is case detection by smear microscopy and treatment of identified cases by the DOTS strategy [4]. A threat to this strategy is the emergence of strains which are resistant to first-line drugs especially isoniazid (INH) and rifampicin (RIF). Such cases may either not be cured by the current first-line treatment regimen or have a more expensive and long treatment course [5]. The tendency to acquire drug resistance may be influenced by the genetic and background of the strain [6]–[8]. TB is caused mainly by a group of genetically closely related species and sub-species together referred to as *M. tuberculosis* complex (MTBC); however human TB is caused mainly by *M. tuberculosis* sensu stricto (MTBss) and *M. africanum*. Based on biochemical analysis, *M. africanum* used to be subdivided into two separate groups. However, genetic analyses have now indicated that *M. africanum* II, predominant in East-Africa is actually a variant of *M. tuberculosis*. In this manuscript, *M. africanum* is defined as the one originally termed *M. africanum* 1 based on biochemical analysis, which is genetically sub- divided into West-African genotype 1 and II. While *M. tuberculosis* is globally distributed, *M. africanum* is important cause of human TB in West-Africa. *M africanum* is responsible for up to 40% of pulmonary TB patients in some West-African countries [9]–[11]. DNA-DNA hybridisation and multi-locus sequencing analysis indicates that the members of MTBC share high genomic similarities [12]. In spite of this, various genetic methods have been developed for strain typing which have been helpful for answering various epidemiological questions and shed new light on the biology of the pathogen. Short- and long-term epidemiologic questions such as describing transmission dynamics, identifying groups most at risk and risk factors for transmission, estimating recent-versus-reactive disease and the extent of exogenous re-infection have been addressed using these methods [13], [14]. Genotyping of MTBC has also helped in tracking the transmission links between individuals, and demonstrated instances in which epidemiologically linked people were in fact infected with unrelated strains [13], [15], [16]. Molecular methods that have been employed for strain differentiation among MTBC include Restriction Fragment Length Polymorphism (RFLP) analysis [9], spoligotyping [17] which detects variability within the direct repeat locus, Variable Number Tandem Repeats (VNTR) [18] and large sequence polymorphism (LSP) analysis [11]. While VNTR and spoligotyping is usually used for transmission and phylogenetic studies, LSP analysis is used for species- and sub-species differentiation of MTBC and for phylogenetic analyses [9], [13].

To date, only one study has used spoligoytping to study the population structure of MTBC causing human TB in Ghana [19]. This study reports the use of molecular methods to analyse a set of isolates cultured from sputum samples obtained from pulmonary TB patients attending various health facilities in two regions of Ghana.

## Methods

### Specimen Collection and Patients' Characteristics

Specimens included in this study were collected over a period of 17 months (from October 2007 to March 2009) from sputum AFB-positive pulmonary TB cases attending four main health facilities; Agona Swedru Government Hospital, Winneba Government hospital, and St Gregory Catholic Clinic at Budumbura refugee camp,) covering three different districts in the Central region and Effia-Nkwanta regional hospital in the western region of Ghana before they were put on anti-TB drug. After informed consent was obtained, two sputum specimens were collected from each individual, and a structured questionnaire was used to obtain standard demographic and epidemiologic data on patients. The sputum specimens were either mixed with 1% cetylpyridinium chloride and transported within seven days of collection to the laboratory at the NMIMR or stored in a fridge and transported within 72 hours of collection on ice for Petroff decontamination before cultivation [20]. Ethical clearance for the study was approved by the institutional review board of the Noguchi Memorial Institute for Medical Research (Federalwide Assurance number FWA00001824). The procedure for sampling in this study was mainly the same as those used in routine management of TB in Ghana. However, informed consent (written in the case of literate participants and oral for those who cannot read) was sought from all participants before their inclusion in the study. In the case of children below sixteen years, informed consent was sought from their parents or guardians. The objectives and benefits of the study were explained to them all. They were assured of the confidentiality of all information collected from them. Inconveniences of participation were explained to the participants and they are free to join the study or exit at any time which will not in any way affect their treatment.

### Mycobacterial Isolation

All specimens after decontamination were cultured on two Lowenstein-Jensen slopes; one with supplemented with 0.4% sodium pyruvate to enhance the isolation of *M. africanum* and *M. bovis*. The cultures were incubated at 37°C and were read weekly for growth for a maximal duration of 16 weeks.

Preliminary identification of suspected mycobacterial isolates was done by AFB staining and biochemical methods such as susceptibility to p-nitro benzoic acid (PNB) and to thiophene carboxylic acid hydrazide (TCH), pyrazinamidase activity (PZA), nitrate reduction, niacin production [20].

### Drug Susceptibility Testing

The susceptibility pattern of all identified mycobacterial isolates to isoniazid (0.2 µg/ml), rifampicin (40 µg/ml), streptomycin (4 µg/ml), and ethambutol (2 µg/ml) for all *M. tuberculosis* complex primary isolates was determined phenotypically by the indirect proportion method on L–J slants, as described previously [21]. Drug resistance was expressed as the proportion of colonies that grow on drug containing medium to drug-free medium and the critical proportion for resistance was 1% for all drugs.

### DNA Extraction

DNA extraction was done according to previously outlined protocol [22]. About two- 5 µl loop full of bacteria were heat killed in 500 µl of an extraction mixture (50 mM Tris–HCl, 25 mM EDTA, and 5% monosodium glutamate). After cooling, 100 µl of a 50 mg/ml lysozyme solution was added and incubated with shaking for 2 h at 37°C. Sixty micro litres of 20 mg/ml proteinase K solution in a 10× buffer [100 mM Tris–HCl, 50 mM EDTA, 5% sodium dodecyl sulphate (pH 7.8)] were then added and incubated at 45°C overnight. The bacterial cell wall was fully disrupted by adding 200 µl of 0.1 mm-diameter zirconia beads (BioSpec Products) to each sample and vortexing at full speed for 4 min. Beads and undigested tissue fragments were removed by centrifugation at 14,000 rpm for 3 minutes, and the supernatants were transferred to fresh tubes for phenol-chloroform (Fluka) extraction. The DNA contained in the upper phase was precipitated with ethanol and re-suspended in 100 µl of water.

### Genotyping

Genotyping of MTBC isolates was done in a stepwise mode (table 1). All isolates included in the study were first identified as belonging to MTBC by PCR targeting the insertion sequence *IS6110* as previously described [23]. Species were defined by analysing for large sequence polymorphisms (LSP) at the regions of difference (RD) 9, 12 and 4 using published flanking primers [9], [10]. Isolates that were identified as *M. africanum* were further typed for RD702 and RD711; and the Cameroon lineage, which we assumed to be the most dominant among the MTBss was defined by a deletion in RD726 also using flanking primers [10], [11]. All the isolates that we confirmed as *M. tuberculosis* complex were further typed by spoligotyping [17]. Briefly the direct repeat region of each genome was amplified using primers DRa (59-CCG AGA GGG GAC GGA AAC-39) and biotinylated Drb (59-GGT TTT GGG TCT GAC GAC-39). The amplified DNA was tested for the presence of specific spacers by hybridization with a set of 43 oligonucleotides derived from the spacer sequences of *M. tuberculosis* H37Rv and *M. bovis* BCG P3 (the GenBank accession no. for the sequence of *M. tuberculosis* H37Rv is Z48304, and that for *M. bovis* BCG P3 is X57835). Bound fragments were revealed by chemiluminescence after incubation with horseradish peroxidase-labeled streptavidin (Boehringer Mannheim). In order to prevent cross contamination, PCR amplifications and pre-PCR procedures were conducted in physically separated rooms. Negative water controls were PCR amplified and included on each blot to identify any possible amplicon contamination. In addition, Positive controls (H37Rv and *M. bovis* BCG DNA) was amplified and included on each blot.

**Table 1:** PCR Procedures used for species and lineage identification of *M. tuberculosis* complex isolates obtained in this study.

	Locus Analyzed						
*M. tuberculosis* complex	IS6110	RD4	RD9	RD12	RD702	RD711	RD726
*M. tuberculosis* OTCF	+	+	+	+	nd	nd	+
*M. tuberculosis* CF	+	+	+	+	nd	nd	−
*M. africanum* WAFri I	+	+	−	+	+	−	nd
*M. africanum* WAFri II	+	+	−	+	−	+	nd

PCR polymerase chain reaction; RD =  regions of difference;

+ =  locus intact; − =  locus deleted

OTCF =  Other than Cameroon family; CF = Cameroon family

WAfri = West-African type. nd = not determined.

### Data Analysis

Spoligotypes were analysed as character types. The obtained spoligotyping patterns were compared with those available in the international spoligotype database (SpolDB4) [24] containing 35,925 spoligotypes comprising 39,295 isolates from 122 countries. A shared type was defined as a spoligotyping pattern common to at least two isolates, and clades were assigned according to signatures described in the database. Phylogenetic relationships among the isolates were inferred from Spoligotyping using the MIRU-VNTR plus software. In addition we compared the diversity within the main lineages that is MTBss and *M. africanum* I as well as between the main sublineages *M. africnaum* West-African type I (WafrI) and the Cameroon family (Euro-American). This was done by comparing both the number of isolates and the number of different spoligotype patterns between these groups. The significance difference among different categories of specific demographic character as well as drug resistance and isolate lineage were analysed by the chi squared test and Fisher's exact test as appropriate using STATA, and the medians of the ages of the various groups were analysed by Mann-Whitney U test.

## Results

### Study Population and Bacterial Samples

One hundred and sixty-two isolates representing 70% of isolates (162/232) obtained from sputum samples consecutively collected from patients suffering from pulmonary TB attending four main health facilities in the Central and Western regions of Ghana were analysed. Age of patients enrolled ranged from 2 to 90 years, with a median age of 38.5 years. Out of the 162 cases, the nationality of 160 was indicated. 12 were Liberians, two Togolese and 1 each of, Nigerians and Ivories, respectively living in the Bujumbura refugee camp. The remaining 144 patients were Ghanaians. Of the 161 TB cases who indicated their sex, 109 (67.7%) were male while 52 (32.3%) were female.

### Prevalence of the different sub-species and lineages within the *M. tuberculosis* complex by LSP analysis

**Figure 1:**
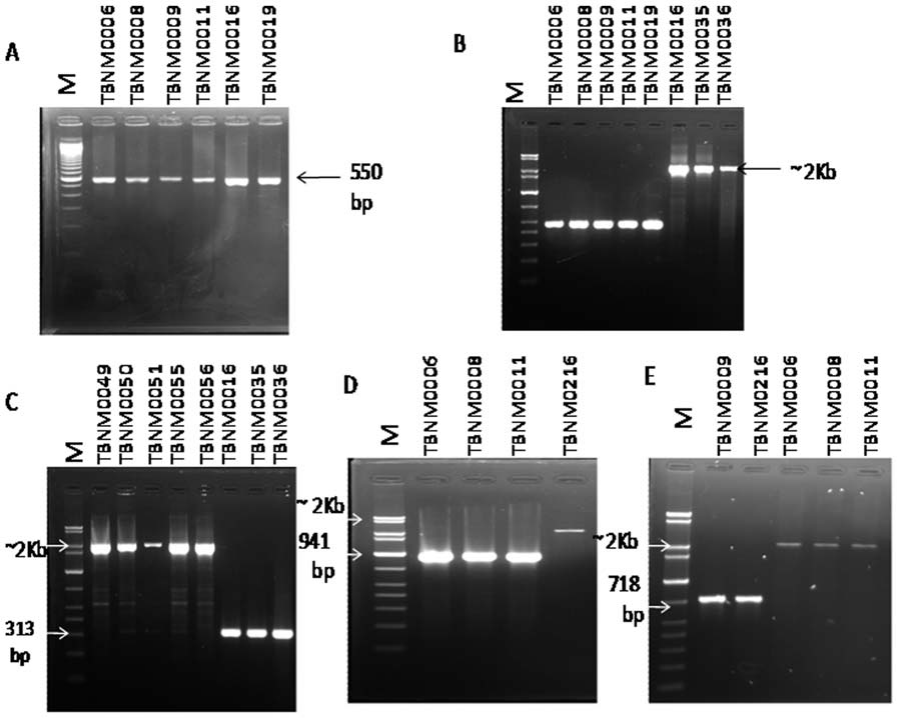
Polymerase chain reaction procedures used for the differentiation of the MTBC Amplicons obtained after various PCR analysis performed in the study. A) *IS6110;* B–E =  Large sequence polymorphism analysis of different regions of difference (RD) RD9 (b), 726(c), 711 (d) and 702 (e) showing deleted and intact genomic regions at the respective locus.

A total of 162 isolates were confirmed as belonging to MTBC (Table 2). All isolates had the insertion sequence IS*6110* evident by the production of the specific 550 bp amplicon corresponding to a portion of the IS*6110* DNA sequence (Figure 1a). The presence of the main lineages within MTBC were analysed by large sequence polymorphism analysis at various regions of difference (RD). RD9 analysis by PCR identified 130/162 (80%) of the isolates as MTBss defined by the detection of an intact PCR product (Figure 1b), and among this group, RD726 PCR (Figure 1c) defined 59/130 (45%) as belonging to the Cameroon sub-lineage. 32/162 (20%) were classified as *M. africanum* type I by analysis of the RD9 region (Figure 1b); the majority of them 26 (81%) were identified by RD711 PCR (Figure 1d) as West-African I, and 6 (19%) as West-African II by RD702 PCR (Figure 1e). Based on RD12 and RD4 analyses, no *M. bovis* was detected.

**Table 2:** The level of resistance obtained from the main lineages and sub-lineages that were tested in the study.

Tested Drug	*M. tuberculosis (n = 92) N (%)*	*M. africanum (32) N (%)*	*P value* *
STR	35 (38%)	4 (12.5.1%)	0.008
INH	14 (15.2%)	2 (6.25%)	0.237
RIF	7 (7.6%)	1 (3.1%)	0.679
EMB	4 (4.3%)	1 (3.1%)	1.000
MDR	4 (4.3%)	0 (0%)	0.572
ANY RESISTANCE	40 (43.5%)	7 (21.9%)	0.030

Tested Drug	Cameroon Sub-lineage *(n = 47) N (%)*	West-African I *(n = 26)* *N (%)*	*P value*
STR	19 (40.4%)	3 (11.5%)	0.015
INH	9 (19.1%)	1 (3.8%)	0.086
RIF	5 (10.6%)	1 (3.8%)	0.412
EMB	4 (8.5%)	1(3.8%)	0.649
MDR	4 (8.5%)	0 (0%)	0.290
ANY RESISTANCE	22 (46.8%)	6(23.1%)	0.046

The resistance was measured by the proportion method.

*Where cells had values 5 or less the P value was computed using the Fisher's exact test.

### Spoligotyping Patterns

One hundred and sixty-one isolates comprising the 31 *M. africanum* and 130 MTBss isolates were spoligotyped, and the different lineages and corresponding spoligotype patterns are indicated in Table 3. Even though we acknowledge limitation in the discriminatory ability of spoligotyping, we defined a cluster as spoligotypes that contained two or more isolates with identical spoligotyping pattern in our analyses. Based on this definition, clusters of between 2 and 41 isolates were observed in this study. In all, 56 distinct spoligotyping patterns were obtained; 39 and 16 different patterns were obtained from the MTBss and *M. africanum* lineages, respectively. 27 different clusters involving 131 out of 161 (81.4%) of the isolates were observed, MTBss were more likely to be in spoligotyping clusters, with 111/130 (85.4%) isolates clustered within 20 different spoligotypes, compared to 21/31(67.7%) of the *M. africanum* isolates grouped in 7 spoligotyping clusters (OR: 2.78, 95%CI = 1.0004–7.35, p = 0.02). A large cluster consisting of forty-one isolates (25.3%) shared a spoligo-pattern defined in the latest spoligotype database (SpolDB4) with SIT number 61; these strains were identified by LSP involving the deletion of RD726 as belonging to the Cameroon sub-lineage.

**Table 3:** Spoligotyping profile for *M. tuberculosis* complex isolates from Ghana as defined by RDs.

^a^RD9	RD711	RD702	RD726	Spoligoprofile^b^	Sub lineage	No of isolates	Spoldb4^c^
**Undel^d^**			del^e^	1111111111111111111111000111111100001111111	Cameroon	41	61
**Undel**			del	1111111111111101111111000111111100001111111	Cameroon	2	115
**Undel**			del	1111111111111111111111000111111100001110111	Cameroon	1	403
**Undel**			del	1111111111111111111111000111001100001111111	Cameroon	6	772
**Undel**			del	1111111111111111111111001111111100001111111	Cameroon	1	1580
**Undel**			del	1111111111110111111111000111111100001111111		2	
**Undel**			del	1111111111111111111111000000111100001111111		3	
**Undel**			del	1111111111111111111110000111111100001111111		1	
**Undel**			del	1111111111110101111111000111111100001111111		1	
**Undel**			del	1111111111111101111111000111111100001101101		1	
**Undel**			Undel	0000000000000000000000000000000000111111111	Beijing	4	1
**Undel**			Undel	1001111000111111111111111111000010111111111	EAI	4	340
**Undel**			Undel	1111111111111111111111011111111100001111111	Ghana	1	44
**Undel**			Undel	1111111111111111111111111111111100001111111	Ghana	13	53
**Undel**			Undel	1111111111111111111111111111111100001001111	Ghana	1	65
**Undel**			Undel	1111111111111111111110111111111100001111111	Ghana	2	86
**Undel**			Undel	1111111111111101111111111111111100001111111	Ghana	1	118
**Undel**			Undel	1111111111111111111111101111111100001111111	Ghana	1	373
**Undel**			Undel	1111111111111111111111111111011100001111111	Ghana	1	462
**Undel**			Undel	1111111111110111111110111111111100001111111	Ghana	2	504
**Undel**			Undel	1111111111111111111111101000000100001111111	Haarlem	2	45
**Undel**			Undel	1111111111111111111111111111110100001111111	Haarlem	6	50
**Undel**			Undel	1111111111111111111111111000000100001110111	Haarlem	1	62
**Undel**			Undel	1001111111111111111111111111110100001111111	Haarlem	1	655
**Undel**			Undel	1111111111111111111111111100000000000111111	Haarlem	3	1498
**Undel**			Undel	1001111111111111111111111000000100001111111	Haarlem	2	1652
**Undel**			Undel	1111111111111111111100001111111100001111111	LAM	2	42
**Undel**			Undel	1111111100001111111111111111111100001111111	S	2	1223
**Undel**			Undel	1110111111111111111111111111111100001110111	Uganda 1	4	848
**Undel**			Undel	1110000000001111101111111111111100001101111	X3	3	70
**Undel**			Undel	1110000000001111101111111111111100001110000	X3	6	200
**Undel**			Undel	1111111111111111101111111111111100001111111	X	1	119
**Undel**			Undel	1101111000000011111100001111111100001111111		2	
**Undel**			Undel	1101111111111111111111111111000010101111111		1	
**Undel**			Undel	1001111111101000011111111111110100001111111		1	
**Undel**				1111111000011111111111111111000010111111011		1	
**Undel**				1000000000001111111111000000000000111111111		1	
**Undel**				0000000000000000000000001111111111110000111		1	
**Undel**				1001111000111011111111111111000000111111111		1	
**Del**	Del	Undel		1011111000001111111100001111111111110001111	West African I	7	319
**Del**	Del	Undel		1111111000001111111100001111111111110001111	West African I	4	331
**Del**	Del	Undel		1111111000001111111111111111111111110001111	West African I	2	428
**Del**	Del	Undel		1111111000001111111100001111111101110001111	West African I	1	
**Del**	Del	Undel		1111111000001101111000001111111111110001111	West African I	1	
**Del**	Del	Undel		1111111000000111111100001111111111100001111	West African I	1	
**Del**	Del	Undel		1111111000001111111100001111111111100001111	West African I	2	
**Del**	Del	Undel		1101111000000111111100001111111111110001111	West African I	1	
**Del**	Del	Undel		1111111000001111111100000100000000000000000	West African I	3	
**Del**	Del	Undel		1111110000001111111100000000000000000001111	West African I	1	
**Del**	Del	Undel		1011111000001001111100001111111111110001111	West African I	2	
**Del**	Del	Undel		1111111000000111111100001111111111110001111	West African I	1	
**Del**	Undel	del		1011110001111111111111111111111111111101111	West African II	2	318
**Del**	Undel	del		1111110001111111111111111111111111111101111	West African II	1	181
**Del**	Undel	del		1111110001111111111111111111111100000001111	West African II	1	
**Del**	Undel	Del		1111110001111111111110001111111111111100111	West African II	1	

^*a*^RD: Regions of difference.

^*b*^1, presence of the spacer; 0, absence of the spacer.

^*c*^Spoldb4 are the coded patterns in the international spoligotype database.

*^d^*Undel: Undeleted, ^*e*^Del: Deleted.

Comparing our isolate patterns with the SpolD4 database, 130/161 (80.7%) isolates had previously defined shared spoligotype numbers; while the remaining 31 isolates had unidentified patterns. 14 of the isolates which gave newly identified spoligotypes clustered into six groups of between 2 and 3 isolates. The remaining 17 isolates gave unique patterns.

In addition to the Cameroon family, 8 additional spoligotyping families were identified among the MTBss isolates that we tested. These are 15 isolates (11.53%) belonging to the Haarlem family, 22 isolates of the Ghana family (16.92%), 4 isolates (3.08%) each of “Beijing”, Uganda I and EAI, respectively, LAM (2∶1.54%), S (2∶1.54%) and X (1∶0.77%). 9 isolates had SIT numbers with no identified sublineages while 16 had no SIT numbers.

### Prevalence of drug resistance among the main lineages and sublineages

The drug susceptibility patterns of 92 of the 130 MTBss isolates and all the *M. africanum* isolates were analyzed by the proportion method. Table 2 specifies the level of resistance that was obtained among the main lineages and sublineages analysed in the study. While we did not find any difference in resistance to INH, RIF and EMB, we found that MTBss (OR = 4.30, CI95% 1.33–18.10, p<0.008) and the Cameroon sub-lineage (OR = 5.20, CI95% 1.27–30.22p<0.015) were more likely to be STR resistant when compared to all *M. africanum* and the West-African I sublineage respectively. Overall, the proportion of MTBss isolates resistant to any of the tested drugs was higher when compared to all *M. africanum* (OR = 2.74, CI95% 1.01–8.24, P<0.03).

### Epidemiological Associations

Table 4 shows some demographic parameters we analysed. The median age of 48 female participants who indicated their age (29.8, range = 2–90)) was lower but not statistically significantly different from that of male participants (median = 41, range = 18–73). There was no significant difference in median age of participants from whom *M. africanum* was isolated (median = 42, range = 16–68) compared to that of MTBsss (median = 38.5, range = 2–90). Female and male TB patients were equally likely to carry MTBss as opposed to *M. africanum*. 11 out of the 16 foreigners (68.8%) were male and only five were females, while 67.4% of the Ghanaians were males.

**Table 4:** Demographics and main lineages of *M. tuberculosis* complex isolated from participants from whom sputum samples were analysed.

Parameter	Frequency n (%)
**Sex**	
Males	109 (67.7%)
Females	52 (32.3%)
**Nationality**	
Ghanaian	144 (90.0%)
Liberian	12 (7.5%)
Other West-African Nationals	4 (2.5%)
**Nationality and Sex**	
*Ghana*	
Females	47 (32.6%)
Males	97 (67.4%)
*Foreigners*	
Females	5 (31.3%)
Males	11 (68.8%)
***M. africanum***	
Males	20 (64.5%)
Females	11 (34.5%)
***M. tuberculosis***	
Males	89 (68.5%)
Females	41 (31.5%)
***M. africanum***	
Mean age	39.8±15.3
Range	16–68
Median	42
***M. tuberculosis***	
Mean Age	39.7±15.7
Range	2–90
Median	38.5

## Discussion

This study sought to use various molecular methods in an African setting for the characterisation of MTBC isolates obtained from TB patients attending various health facilities. Three main methods which were used in this study namely, IS*6110* PCR, RD-PCR analysis and spoligotyping this also makes our study the first to be conducted in which the same sets of isolates from Ghana are analysed by RD-PCR and spoligotyping. This will provide the basis for the design and implementation of in-depth molecular epidemiological studies in the country in future.

MTBC lineages that affect humans have been subdivided into six geographically linked phylogenetic lineages defined by both SNPs and LSP analysis [11], [12]. When Gagneux *et al* analysed a collection of 875 MTBC isolates from patients originating from 80 countries using LSP analysis, one of the major observations was that two of the six lineages are dominantly found in West-Africa; West-Africa I and West-Africa II. West-Africa I is predominantly found around the Gulf of Guinea and West-Africa II is prevalent in western West-Africa [25].

Our LSP analysis of 162 MTBC isolates from Ghana revealed that 20% belonged to *M. africanum*. Eighty-one percent of *M. africanum* isolates belonged to West-Africa I and 19% to West-Africa II. *M. africanum* was first identified in 1968 in Senegal and was described biochemically as having characteristics between *M. tuberculosis* and *M. bovis* [26]. *M africanum* has been found in some studies to cause up to 40% of human TB in West-Africa [25]. The observed percentage in the current study is higher than in a previous study, which found *M. africanm* type I to be up to 13% [27]. However, in that earlier study, mycobacterial characterization was based solely on biochemical methods. In our analysis we found isolates with discordant results between the biochemical analysis and the molecular identification we established (data not reported here). For example some of the isolates that tested positive for pyrazinamadase and negative for niacin accumulation were found to be *M. tuberculosis* rather than *M. bovis*. These discordant findings were clarified by the RD-PCR analysis. This shows that reliance on biochemical methods for species differentiation is not only cumbersome but can also lead to mis-classification [28]. We therefore suggest that reference laboratories in endemic countries should establish genetic identification systems to confirm results of biochemical differentiation methods or abandon biochemical differentiation altogether. Also in Senegal it has been observed, that the proportion of *M. africanum* causing TB varies by region [29]. The same may be true for Ghana, as the current study was conducted in the Central region of Ghana, while in the previous study isolates from the Greater-Accra region were analysed [27]. The proportion of *M. africanum* West-African I lineage (>80%) of the total *M. africanum* isolates found in this study is high compared to the study reported by Goyal et al [19]. in which out of the 75 isolates whose pattern was indicated, 26% were *M. africanum* and of this only 52% belonged to the *M. africanum* West-African I lineage. The previous study collected samples from the Ashanti region which is in the north central part of the country whilst the current study was conducted in the south-western part of Ghana. This disparity could also confirm that even within *M. africanum* endemic countries; there are regional variations in distribution. However, this need to be evaluated further in a population-based study as the sample sizes in both studies is small. The reason why *M. africanum* is common among MTBC isolates in humans in West-Africa but essentially absent in the rest of the world needs to be investigated further [25].

The outcomes of TB infections in humans are extremely variable, ranging from lifelong latent infection to active disease with variable degrees of extra-pulmonary involvement. In addition to host and other environmental factors, this variability could be the result of genetic variation in infecting strains. There is increasing evidence from experimental studies that points the MTBss lineages differ in virulence and immunogenicity [30]. It has been suggested that *M. africanum* is less virulent than MTBss, since a study in The Gambia demonstrated that although MTBss and *M. africanum* infected cases were equally able to transmit infections to household contacts, more contacts infected with MTBss progressed to active disease [31]. In this work we evaluated the effect of strain genetic background and the occurrence of drug resistance by comparing the proportion of phenotypic drug resistance between the different MTBC lineages. We found that MTBss was more likely to be resistant to any of the tested drugs when compared to *M. africanum*, this association was primarily driven by resistance to STR. Drug resistance has been often associated with the Beijing lineage for reasons that remain unclear [32]. Our finding that *M. africanum* was less likely to be resistant to STR suggests putative interaction between drug resistant and strain genetic background. There is mounting evidence that different lineages of MTBC can be associated with different drug-resistance conferring mutations [7], [32], perhaps indicating an interaction between the strain genetic background and particular drug resistance mutations [33]. A study conducted in Ghana using DNA sequencing detected significant variations in the proportion of INH resistance-conferring mutations in different MTBC lineages. While there was a significantly higher proportion of *katG* 315 mutations in MTBss, *M. africanum* West-African I strains were more likely to harbour a mutation in the promotor region of *inhA* [6]. Future work in our laboratory will try to confirm these results.

Among the 161 isolates that we analysed by spoligotyping, 56 distinct spoligotypes were identified, indicating a wide diversity among isolates obtained from a small region in Ghana.

We found that MTBss isolates were more likely than the *M. africanum* isolates to be part of a spoligotyping cluster. This observation could indicate an overall higher genetic diversity among *M. africanum* compared to MTBss in Ghana, similar to what has been found in earlier publications from West Africa [9], [31]. This supports the hypothesis that *M. africanum* established itself in West Africa before the Euro-American *M. tuberculosis* lineage was introduced during European exploration and colonization [34]. Alternatively, MTBss might be more transmissible than *M. africanum* in Ghana. However, whether these spoligotyping clusters represent linked transmission events will need to be confirmed by genotyping methods such as MIRU-VNTR which exhibit a higher discriminatory power. MIRU-VNTR typing as well as single nucleotide polymorphism analyses are currently being established in our laboratory in Ghana.

We conclude that molecular methods are more robust and specific than the classical biochemical test for MTBC species determination and that such techniques can and should be established more widely in countries of sub-Saharan Africa. Ghana is one of the few countries which harbour both lineages of *M. africanum* (i.e. West-Africa I and West-Africa II). Given the current efforts in TB vaccine development, strain diversity should be considered when evaluating new vaccine candidates in areas where *M. africanum* is prevalent.
